# Organosolv Lignin from European Tree Bark: Influence of Bark Pretreatment

**DOI:** 10.3390/ma14247774

**Published:** 2021-12-16

**Authors:** Jakub Grzybek, Thomas Sepperer, Alexander Petutschnigg, Thomas Schnabel

**Affiliations:** 1 Forest Products Technology and Timber Constructions Department, Salzburg University of Applied Sciences, Markt 136a, 5431 Kuchl, Austria; jakub.grzybek@fh-salzburg.ac.at (J.G.); alexander.petutschnigg@fh-salzburg.ac.at (A.P.); 2 Department of Wood Science and Technology, Faculty of Forestry and Wood Technology, Mendel University in Brno, Zemědělská 3, 613 00 Brno, Czech Republic; 3 Salzburg Center for Smart Materials, Jakob-Haringer Straße 2a, 5020 Salzburg, Austria; 4 Department of Material Sciences and Process Engineering, University of Natural Resources and Life Sciences (BOKU), Konrad Lorenz-Straße 24, 3340 Tulln, Austria; 5 Faculty of Furniture Design and Wood Engineering, Transilvania University of Brasov, B-dul. Eroilor nr. 29, 500036 Brasov, Romania

**Keywords:** byproduct, valorization, tannin, polyphenol, extract, circular economy

## Abstract

As lignin is becoming more and more attractive to industry and the circular economy continues to grow, the utilization of a byproduct that, to date, has been underrated by the wood industry is investigated as an abundantly available source of lignin. Bark from spruce, larch and beech tress is extracted using the organosolv process with and without prior hot water extraction. The influence of the treatment on chemical properties of the lignin was determined by spectrophotometric, chromatographic, and vibrational spectroscopy. It was found that hot water extraction prior to organosolv extraction influences the chemical composition, antioxidative properties and molecular weight distribution of the obtained extracts. While hot water extracts are rich in flavonoids, organosolv fractions can contain high amounts of organic acids depending on whether they are from a hardwood or softwood source. This investigation lays the foundation for further research into the utilization of byproducts to generate high-value resources.

## 1. Introduction

Lignin is a natural polyaromatic macromolecule found in the cell walls of different plants. In recent years, research on lignin has gained more and more attention because the properties of lignin, such as its reactivity, make it suitable for the synthesis of biopolymers, including bioplastics or lightweight materials [1,2].

**Figure 1:**
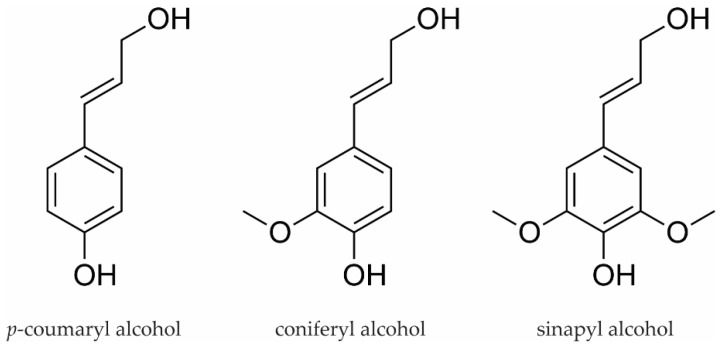
Main building blocks of lignin.

Lignin is derived from phenolic monolignols or hydroxy cinnamyl alcohols. The process involves the biosynthesis of lignin monomers, which are polymerized by peroxidase and laccase to plant cell walls. These units, after polymerization, are guaiacyl (G) from coniferyl alcohol, syringyl (S) from sinapyl alcohol and *p*-hydroxyphenol (H) from *p*-coumaryl alcohol differing by 3,5-substitution with zero to two methoxy groups (Figure 1). The composition of the units varies in different plant species. For example, the lignin of softwoods contains predominantly G units and only a few H units, while the lignin of hardwoods consists mainly of G and S units. Grasses, on the other hand, have a larger proportion of H units [3,4]. Lignin in its native state has, to date, not been extractable. There are various ways to separate lignin from cellulose and other components of plants, depending on the desired target product. In pulp and paper production, for example, lignin is considered a disturbing element and is completely removed from the wood to obtain the purest possible cellulose. The most common processes used in the paper industry, such as the sulfite process, alter the lignin, making it unsuitable for further processing. In this case, the so-obtained lignosulfonate is usually used for combustion [5]. An alternative to this process is organosolv extraction, which can hydrolyze lignin with high chemical activity (preserving the reactive hydroxy groups) and purity using an organic solvent such as ethanol, as well as a high temperature (up to 250 °C) and high pressure during the extraction [6,7].

Most research on organosolv lignin deals with extraction from wood and annual or perennial plants [8,9,10]. Growing plants to extract lignin is not economical, and many plants are in direct conflict with the food industry. Some solutions for sustainable and more economical extraction of lignin are biomasses, i.e., various plant wastes that have no use other than combustion. One potential alternative source of lignin is tree bark, which is currently underestimated as a valuable resource and in practice is widely considered a byproduct from the debarking of wood for further processing [11]. Only a minor part of this bark is presently used on a small scale e.g., in horticulture as mulch. The valorization of tree bark has the potential to more efficiently use resources, with both ecological and financial benefits [12,13]. Bark differs from wood by its high content of extractives as well as its high amount of inorganic material [14]. Currently, only certain bark extracts, mostly the polyflavonoid tannins, are commercially extracted and used. Tannins have been researched for polymer formulations such as adhesives [15], lightweight rigid foams for thermal and acoustic insulation [16,17], and wastewater treatment [18,19,20].

Industrial condensed tannin is obtained from wood species in the Australian hemisphere (Brazil, Argentina, Tanzania, and Australia). The long distances of transportation to Europe and the uncontrolled afforestation in these regions make it difficult to use tannin in Europe as a bioresource [21]. Therefore, the extraction of lignin from the bark of European wood species, especially from the most widely used such as spruce, beech, and, in the Alpine regions, larch, could both create a good alternative to tannin and use the residual waste—bark—in a new way. This would not only create higher added value for the wood processing industry but also contribute to the further development of biomaterials.

In studies conducted previously on the topic of extracting lignin from tree bark using the organosolv process, Liu et al. [11] proved that valuable crude lignin can be obtained from pine and oak bark. Unkelbach et al. [13] performed a single-stage ethanol extraction of lignin on spruce bark. Due to its high concentration of non-lignin extractables, the obtained lignin is impure. Therefore, in this study, the influence of hot water pretreatment on the yield, chemical composition, and average molecular weight of organosolv lignin obtained from three European tree barks, namely spruce, larch and beech, is investigated.

## 2. Materials and Methods

### 2.1. Materials

Fresh wood bark from larch (*Larix decidua*), spruce (*Picea abies*) and beech (*Fagus sylvatica*) was kindly provided from local sawmills in Kuchl (Austria), which was manually debarked and collected. Reagents, such as sulfuric acid (96%), ethanol (99%), 1,4-dioxan, sodium carbonate, tetrahydrofuran were purchased from Carl Roth (Karlsruhe, Germany). Methanol and dimethyl sulfoxide were obtained from VWR (Rue Carnot, France). Folin–Ciocalteau reagent and hydrochloric acid (32%) were obtained from Merck (Darmstadt, Germany). All chemicals were used as received without further purification.

### 2.2. Bark Preparation

For all extractions, the chipped barks were dried at 60 °C, then stored at 20 °C and 65% relative air humidity until further operations. Dried barks were ground to particles smaller than 1 mm using a cross beater mill (Retsch, Haan, Germany) and then sieved. A fraction between 63 µm and 1000 µm was collected and used for the extractions.

### 2.3. Extraction

Extraction of water-soluble extracts from barks was carried out in water containing 3% of sodium sulfite of a dry wood bark mass, with a dry solid to liquid ratio of 1:7 (*w/w*). The aqueous solution with 20 g bark powder was heated to approximately 90 °C for 3 h under reflux condenser. After the treatment bark was filtrated and washed three times with 50 mL of deionized water. The solid residue was dried at 60 °C to weight constant. From the liquid part after the rotary evaporator, the crude tannin was recovered. For each bark, extraction was made in triplicate.

Ethanol organosolv lignin (EOL) was extracted from untreated barks as well as from the barks after hot water extraction (HWE). 10 g of bark powder was mixed with 65% ethanol (*v/v*) in a ratio of 1:7 (*w/w*), using sulfuric acid (96%) in 1% of the bark mass (*w/w*) as a catalyst. The treatment was carried out in a custom-built stainless steel reactor at 160 °C for 1.5 h. After cooling down the reactor, the bark was filtered and washed three times with 50 mL of 65% ethanol at 60 °C. The solid residue was washed with water and then dried at 60 °C until constant weight. From the liquid part, the ethanol was diluted with rotary evaporator and a total recovery of ethanol of 70% was achieved. To the solution, 200 mL of deionized water was added and the pH was decreased to two with hydrochloric acid (32%). The precipitating lignin was centrifuged off for five minutes, washed five times with deionized water, and dried at 60 °C. For each bark, extraction was made in triplicate.

### 2.4. Extract Characterization

Total phenolic content (TPC) was measured by the Folin–Ciocalteu assay for all extracts as described elsewhere [22]. 200 µL of the extract solution was mixed with 3000 µL deionized water, 500 µL Folin–Ciocalteu reagent and 2000 µL sodium carbonate (20%). The samples were kept in the dark for 1 h. Afterwards, absorbance at 745 nm against a blank measured using a Shimadzu UVmini 1240 spectrophotometer (Shimadzu, Japan). Water-soluble extracts were dissolved in water and the lignin samples in dimethyl sulfoxide (DMSO) at concertation of 1 mg/mL. Three samples of each fraction were prepared, and duplicate measurements were performed.

Determination of the antioxidant activity was done as described by Alzagameem [23]. The extracted samples were dissolved in water or the lignin samples in 90% dioxane at a concentration of 1 mg/mL. 30 µL of the extract was mixed with 3000 µL of DPPH (6 × 10^−5^ M in methanol). The samples were incubated in the dark for 15 min. The absorbance of the sample (A_15_) was measured against pure methanol as a blank sample at 515 nm. For the initial value (A_0_), the absorbance of the DPPH solution at 515 nm without extract was taken. Three samples of each fraction were prepared, and duplicate measurements were performed of each sample. For the calculation of antioxidant activity as % inhibition following formula (Equation (1)) was used:

(1) $$\%\;inhibition=\frac{A_{0}-A_{15}}{A_{0}}\times 100$$

Attenuated total reflectance–Fourier transform infrared (ATR–FTIR) spectroscopy on untreated barks as well as barks after extractions, water-soluble extracts and lignin was conducted using a PerkinElmer Frontier FT-IR spectrometer equipped with the ATR MIRacle accessory (PerkinElmer, Waltham, MA, USA). The barks and powder extracts were placed on the diamond eye of the instrument and the sample was fixed by tightening the clamp. The samples were analyzed with 32 scans with a resolution of 4 cm^−1^ in the wavenumber range between 4000 and 600 cm^−1^. Each sample was scanned in triplicate and the mean value of these spectra was determined after correction of the baseline and area normalization using the KnowItAll software (John Wiley and Sons, Inc., Hoboken, NJ, USA). Deconvolution of spectra was conducted in the range of 1800 to 1000 cm^−1^. Principal component analysis (PCA) was conducted for the spectra in a range of 2000 to 600 cm^−1^ using the multivariate data analysis function from OriginPro 2021.

### 2.5. HPLC Analysis

RP-HPLC-DAD setup consisted of a degassing unit DGU-20A, a liquid chromatograph LC-20AT, a column oven CTO-10AS with a C18 5 µm column (250 × 4.6 mm I. D.), a refractive index detector RID-20A, and a photodiode array detector SPD-M20A, all of which were manufactured by Shimadzu, Japan. The mobile phase was an isocratic flow of 20% methanol and water with 0.1% TFA at a flow rate of 1 mL/min and an injection volume of 20 µL. Prior to injection, samples were dissolved in 20% MeOH containing 0.1N NaOH to allow for increased solubility of lignin. The liquid extracts from the various extraction processes of the different bark species were used. As reference substances DHBA (dihydroxybenzoic acid), HBA (hydroxybenzoic acid), trans-CA (trans-cinnamic acid), SA (syringic acid), CA (coumaric acid), FA (ferulic acid), V (vanillin) and T (taxifolin) were applied.

### 2.6. Size Exclusion Chromatographie (SEC)

GPC was performed using a Tosho GMHHR-N mixed bed column with tetrahydrofuran as mobile phase. Flowrate was set to 1 mL/min and temperature was kept at 25 °C. A calibration curve was created using polystyrene standards.

### 2.7. Data Curation

If not stated otherwise, data was processed using OriginPro 2021 (OriginLab, Northampton, MA, USA) and BioRad KnowItAll.

## 3. Results and Discussion

### 3.1. Extraction Yields

**Figure 2:**
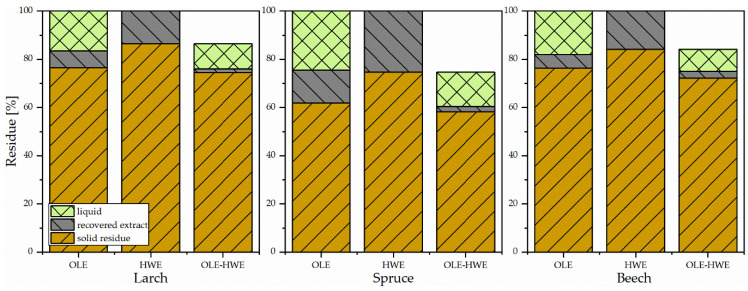
Extraction yield in recovered and liquid form, solid residue.

Distribution of solid residue, recovered extractives, and extractives that remained in the extraction liquid are shown in Figure 2 for the three different processing steps. After the organosolv extraction (OLE) process, water-insoluble lignin was recovered by lowering the pH of the solution, followed by centrifugation. Lignin recovered in this study was centrifuged off from solutions at pH 2. The yield was relatively low compared to the theoretical possible yields (difference in bark weight after and before the organosolv process). When performing ethanol organosolv extraction on bark without pretreatment, between 5.6 (beech) and 13.6% (spruce) of the bark were extracted and recovered as water-insoluble precipitate after centrifugation. Theoretical yield, however, is higher, with 38.2% for spruce, and roughly 23.5% for beech and larch. The gap in recovery can be partially traced back to water-soluble extractives (like tannins) or carbohydrates, which will not be centrifuged off. In particular, spruce bark does contain high amounts of carbohydrates, which explains the much higher theoretical yield [24,25].

Performing hot water extraction prior to the ethanol organosolv process (EOL–HWE), tannins, carbohydrates, and other small molecules are removed from the bark [26]. Again, the higher concentration of sugars as well as the higher amount of tannin in spruce bark led to the highest extraction yield of over 25%, while for other two species had an extraction yield of roughly 16%. Using the hot water–extracted bark residue in the organosolv process, it was possible to extract another 12% of the bark mass from larch, 15% from beech, and 22% from spruce, yet only between 1.3% (larch) and 2.27% (beech) of lignin was recovered from the extraction solution by centrifugation at pH 2. It is possible to increase lignin recovery to 4.5, 7.9, and 2.5% when lowering the extractive solutions pH to 0 for larch, spruce, and beech, respectively. Overall, total mass loss during extraction was the same whether solely performing ethanol organosolv extraction on the bark or pretreating it with hot water extraction. As shown in the next section, the lignin from OLE–HWE process obtained after pre-extraction (HWE) of the bark was less rich in other polyphenolics compared to lignin obtained without prior hot water extraction (only OLE process) of the bark.

### 3.2. Spectroscopic Results

**Figure 3:**
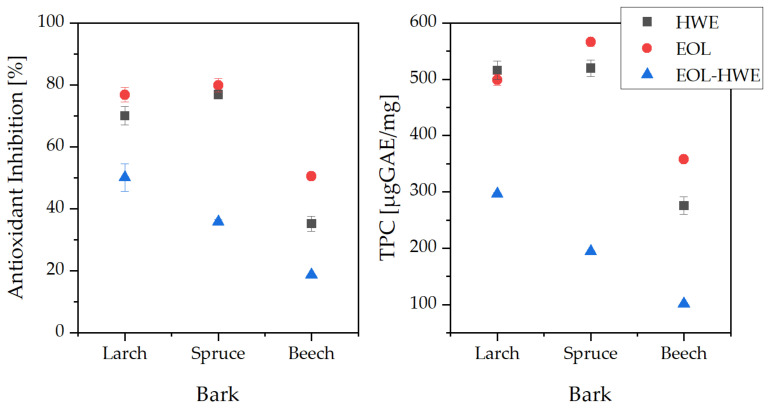
Antioxidant inhibition (**left**) and total phenolic content (**right**) of bark extracts. Standard deviation indicated by error bars.

Extracts were analyzed for their total phenolic content using the Folin–Ciocalteu method. Results are expressed as µgGAE/mg extract and presented in Figure 3 (right). As expected, the water-soluble fraction (hot water extract, HWE), did show a significantly higher content of phenolics in the coniferous species (516 and 520 µgGAE/mg extract, for larch and spruce, respectively) than the extracts from beechwood bark (275 µgGAE/mg extract). Based on dry bark weight, spruce extract did show the highest concentration of phenolics (139.38 µgGAE/mg dry bark), followed by larch bark (87.23) and beech bark (43.62). When only performing organosolv lignin extraction to obtain ethanol organosolv lignin (EOL), TPC for spruce and larch bark did not change significantly. During OLE, some portions of the hot water extractable tannins were also extracted along with the lignin (as seen in Figure 2), explaining the negligible changes in phenolic content. Yet, as the overall extraction yield was lower, TPC/dry bark was also smaller (about half as much for all bark extracts), thus the trend observed during HWE was still visible (spruce > larch > beech). When performing HWE prior to OLE, the overall phenolic content is greatly decreased, again due to the lack of soluble polyphenolics (tannins) in the residue bark after HWE.

Antioxidant inhibition of the extracts is shown in Figure 3 (Left). A trend similar to the total phenolic content is observed, as most of the phenolic components of the extracts show antioxidative properties. The highest inhibition potential (>80%) was observed for HWE and EOL spruce bark extract, followed by >70% for HWE and EOL from larch bark. All beech bark extracts did show a low antioxidant inhibition (50% for EOL, 35% HWE and 18% for EOL after HWE). Similar values for the antioxidant inhibition for spruce have been reported previously [23]. Once more, the similar values for spruce and larch for EOL and HWE could be explained by the higher concentration of tannins extracted during the organosolv process that would normally be soluble in hot water. Generally, lignin does show a lower antioxidative potential compared to tannins.

**Figure 4:**
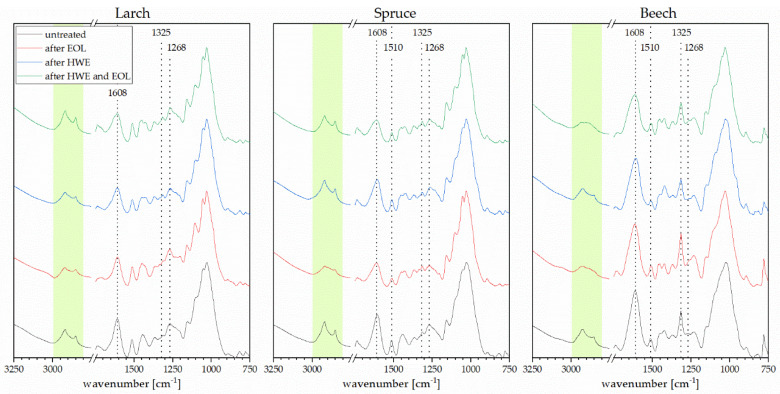
FTIR spectra of unmodified bark and bark after treatment.

**Figure 5:**
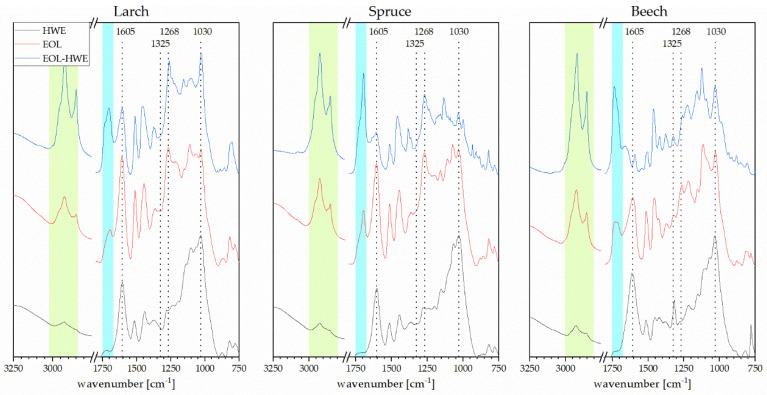
FTIR spectra of the obtained extracts.

ATR–FTIR spectra for bark are shown in Figure 4. For each bark type, a spectrum of the original material is shown, followed by the residues after (i) OLE, (ii) HWE and (iii) and OLE following HWE. For all bark samples, it is visible that HWE alone does not reduce or only slightly reduces the signals at band 2930 and 2855 cm^−1^, corresponding to aliphatic CH, whilst in the organosolv extraction, these signals are reduced completely for beech bark and noticeable for the two coniferous species, suggesting a good extraction of fatty acids and other aliphatic compounds from the bark. When combining hot water extraction with organosolv extraction, the CH signals disappear for beech bark, while they are slightly reduced for spruce. This is also confirmed by the FTIR spectra of the extracts (Figure 5) where peaks corresponding to aliphatic chains are only visible in the organosolv and combined hot water–organosolv fractions. The peak in the region of 1608 cm^−1^ does reduce in intensity for all bark types after extractions. The strongest reduction is observed for spruce bark after OLE and combined HWE and OLE. This is in line with the higher extraction yield for spruce, as this peak represents aromatic skeletal vibrations, found in both tannin and lignin. The peak typically assigned to lignin at 1510 cm^−1^ decreases slightly in spruce and beech samples after each extraction step. Further lignin extraction is confirmed by slight reductions of the peaks assigned to S-type and G-type lignin at 1325 and 1268 cm^−1^, respectively. Once more, the higher extraction yield of spruce explains the more noticeable reduction of the signal in these bands.

As reported previously in literature [18], the hot water soluble fractions do not show any signal in the C=O stretching region of acetyl and carbonyl groups (1700 cm^−1^). This band is more prominent in the organosolv and much more prominent in pretreated organosolv fractions for all three barks, indicating a higher purity of the pre-extracted lignin compared to the purity when solely performing OLE. Peaks and shoulders in the carbonyl region can be attributed to aromatic and aliphatic acetate as well as coumaryl ester [27]. In combination with the signals corresponding to aliphatic skeletal vibrations, the presence of long-chain carboxylic acids is indicated in the lignin extracts, but not in the tannin extracts. The aromatic skeletal vibration signal at 1605 cm^−1^ is more prominent in the organosolv extract without pretreatment than it is in the other extracts, suggesting a mixture of tannin and lignin, while the HWE extract mostly consists of polyphenols and the EOL after HWE contains more phenolic acids and alcohols. This is also supported by the sharper peak at 1030 cm^−1^ usually assigned to CO stretching of phenolic-OH.

S/G ratio is an important identification tool for determining the lignin source as hardwood lignins to have higher concentrations of syringol units, while softwood lignins show higher amounts of guaiacol units. In Table 1, S/G ratio (1325/1268 cm^−1^) is listed for both EOL and EOL after HWE for all three barks. As expected, the different lignin extracts derived from softwood bark showed lower S/G ratios compared to the beech bark derived extracts. After performing hot water extraction of the bark prior to the lignin extraction, S/G ratio for larch and spruce bark extracts are reduced even further, which is another indication for higher purity material, as interference in these regions from other extractives is mitigated.

**Figure 6:**
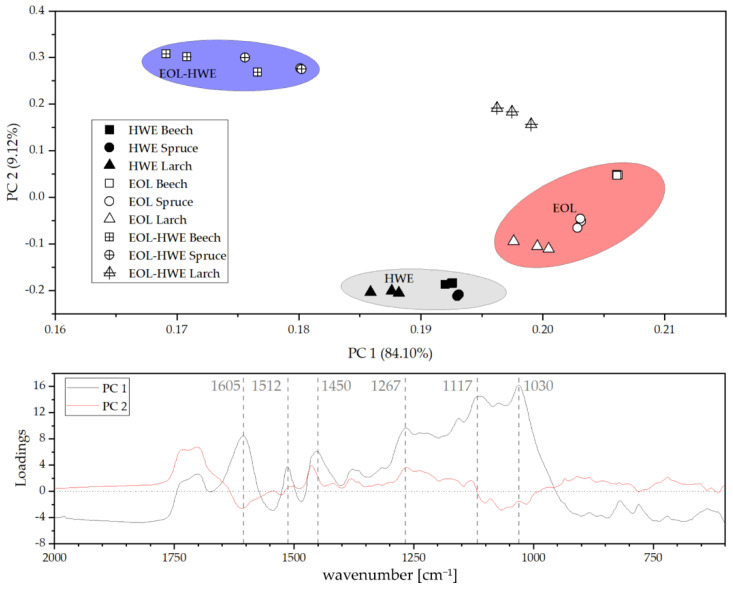
PC 1 vs. PC 2 plot (upper panel) and loadings of PC 1 and PC 2.

Principal component analysis was performed on the 27 spectra from the bark extracts (three bark types, three extracts, three repetitions each). In Figure 6 (upper panel), PC 1 vs. PC 2 are plotted, and three main regions in addition to one outside region can be identified. The first region comprises all hot water extracts from each bark, the second group contains the organosolv extracts without pretreatment, and the third group contains the organosolv extract after HWE for beech and spruce bark. The corresponding larch extract is not associated to the two other bark species. PC 1 accounts for 84% of the variation between the samples and PC 2 accounts for roughly 9%. The loading plot (Figure 6, bottom panel) illustrates further the key differences responsible for the variations. One major influence is the presence of tannins and other extractives present in both the HWE and the organosolv extract without pretreatment as already suggested by TPC determination.

### 3.3. LC-DAD Results

**Figure 7:**
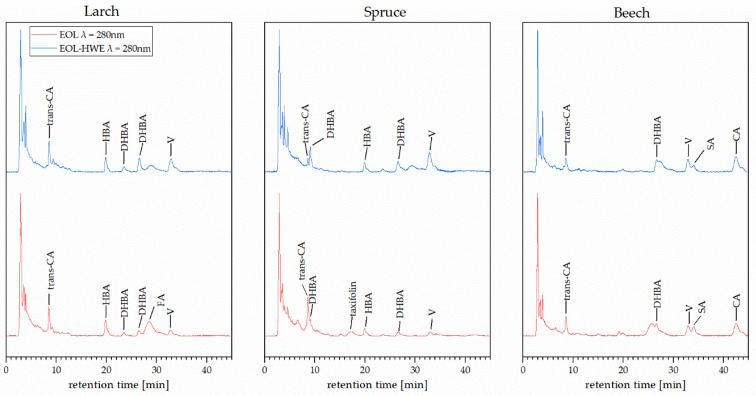
Chromatograms of extracted organosolv lignin with and without pretreatment.

Chromatograms taken at 280 nm using a diode array UV/vis detector are shown in Figure 7 for EOL and EOL–HWE extracts. Peaks identified by matching retention time and/or UV spectrum are labeled with the corresponding compound, family of molecules, or isomer. Relative integrated peak areas of identified compounds are listed in Table 2 including abbreviations.

While the identified peaks from the hot water extracts (HWE) are mostly assigned to flavonoids and flavonoid glycosides, a majority of compounds identified in the organosolv fractions (EOL and EOL–HWE) consist of different organic acids. If no hot water extraction was performed prior to the organosolv treatment, flavonoids are still present in the organosolv extracts for all three barks. As expected, syringic and coumaric acid were most abundant in beech bark lignin, while all of the bark lignins were rich in dihydroxybenzoic acid and isomers thereof.

### 3.4. GPC Results

**Figure 8:**
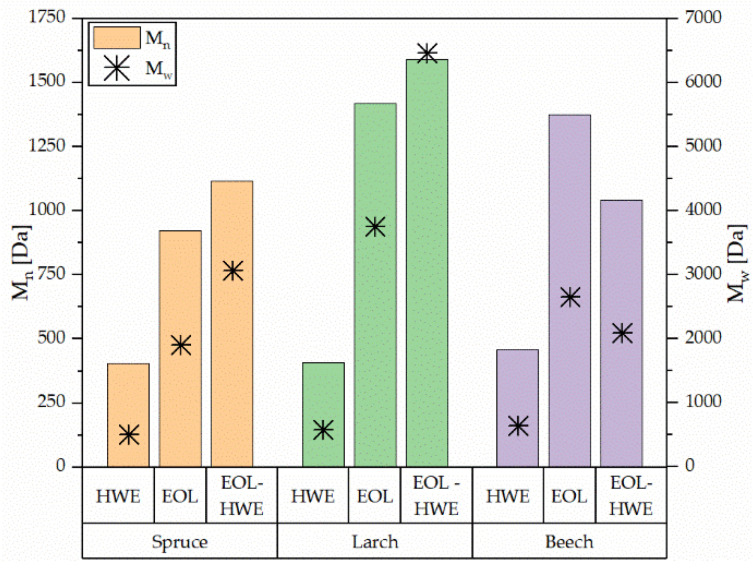
Weight average (Mw) and number average (Mn) molecular mass of obtained bark extracts.

Mass average molecular weight (M_w_) and number average molecular weight (M_w_) as well as polydispersity (M_w_/M_n_) were measured and calculated for all extracts obtained. Results are shown in Figure 8. It is clearly visible that the hot water extracts (HWE) do contain the smallest molecules, in both weight and number average. Weight average lies around 500 Da for spruce and 637 Da for beech. Number average for the two coniferous trees is basically the same, with a weight average of 400 Da. Deciduous beech bark hot water extract does show slightly higher number average molecular weight with 450 Da. These weights are expected as they are in the range of flavonoids, simple phenolics, and glycosides thereof. Polydispersity for all HWE is below 1.5, meaning a homogeneous mixture of same-sized molecules is extracted.

When performing organosolv extraction after hot water extraction, the so-obtained lignin presents the highest M_w_ and M_n_ of the coniferous species, with larch showing the biggest molecules with an M_w_ of 6500 Da and M_n_ of 1600 Da. The smallest level of organosolv lignin is measured for beech bark. However, not all components of the extract were soluble in THF; therefore, bigger molecules may have not been detected. Generally, lignin obtained by organosolv extraction does present a molecular weight in the range of 0.5 to 7 kDa [27,28] and therefore much smaller molecules than other technical lignins, which range up to 50 kDa [29].

Performing organosolv extraction on fresh bark, the obtained extract consists of both low and high molecular mass molecules. The weight average lies between HWE and EOL–HWE, while the number average is comparable to EOL–HWE. Results from GPC once more suggest that higher purity lignin can be obtained by performing hot water extraction on the bark prior to organosolv extraction.

## 4. Conclusions

The influence of hot water extraction pretreatment on the yield, chemical composition, and molecular weight distribution of lignin from European tree bark has been investigated in this comprehensive study. With regard to all different extraction methods, spruce bark provides a greater extraction yield than larch and beech bark. Coniferous extracts are similar in total phenolic content and antioxidant properties.

The results from the FTIR spectroscopy were used to characterize the effects of the three extraction processes of the different bark samples of three European domestic trees. Chemical differences between bark and HWE, EOL, and EOL after HWE-extracted bark were observed and correspond to the functional groups within the lignin structure. Using methods of multivariate statistics, the results demonstrate that classification/clustering of various bark samples with different treatments based on chemical components (e.g., lignin) is possible. Furthermore, some chemical compounds (syringic acid and coumaric acid) could be only determined in the extracts of beech bark, whereas hydroxybenzoic acid was found only in the other extracts of both coniferous barks. The different chemical structure of the hardwood lignin, which can be seen also in the organosolv lignin from beech bark, may contribute to this.

For all three barks, hot water extraction prior to organosolv treatment leads to a low molecular mass water-soluble fraction and a higher molecular mass ethanol-soluble fraction. Without pre-extraction, the obtained lignin does also contain the smaller molecules, which is confirmed by determination of phenolic content and antioxidant inhibition, as this extract presents properties similar to the hot water extracts.

This work showed that hot water extraction prior to organosolv treatment can produce lignin that does not contain flavonoid impurities and is richer in organic acids compared to lignin from not previously treated bark. The information gathered in this investigation provides the foundation for future work in this field and opens up new possibilities for higher quality chemicals obtained from natural resources.

## Figures and Tables

**Table 1:** S/G ratio of investigated bark lignins.

Bark	Extract	S/G ^1^
Larch	EOL	0.42
	EOL–HWE	- ^2^
Spruce	EOL	- ^2^
	EOL–HWE	- ^2^
Beech	EOL	1.26
	EOL–HWE	1.10

^1^ ratio of deconvoluted FTIR band height at 1325 cm^−1^/1268 cm^−1^. ^2^ no peak at 1325 cm^−1^ after deconvolution.

**Table 2:** Relative integrated peak area [%] of identified compounds from HPLC–DAD.

Bark	Extract	DHBA	HBA	Trans-CA	SA	CA	FA	V	T	F
Larch	EOL	3.8	1.7	17.1			18.9	2.5		7.0
	EOL–HWE	9.0	1.2	14.5				14.3		
	HWE									78.9
Spruce	EOL	10.2	0.7	23.2				4.0	9.5	5.3
	EOL–HWE	2.5								
	HWE									78.6
Beech	EOL	3.7		14.8	7.9	9.9	5.8	10.7		10.5
	EOL–HWE	7.8		12.4	4.7	16.5		15.4		
	HWE									74.9

Different isomers of DHBA (dihydroxybenzoic acid), HBA (hydroxybenzoic acid), trans-CA (trans-cinnamic acid), SA (syringic acid), CA (coumaric acid), FA (ferulic acid), V (vanillin), T (taxifolin), F (other flavonoids).

## Data Availability

Not applicable.
